# Use of an ingredient-based analysis to investigate a national outbreak of Escherichia coli O157, United Kingdom, July 2016

**DOI:** 10.2807/1560-7917.ES.2018.23.26.1700627

**Published:** 2018-06-28

**Authors:** Daniel Gardiner, Maya Gobin, Neville Q Verlander, Isabel Oliver, Jeremy Hawker

**Affiliations:** 1 United Kingdom Field Epidemiology Training Programme, Public Health England, London, United Kingdom; 2 Field Epidemiology Service, National Infections Service, Public Health England, Bristol, United Kingdom; 3 Statistics, Modelling and Economics Department, National Infection Service, Public Health England, London, United Kingdom; 4 Field Epidemiology Service, National Infections Service, Public Health England, Birmingham, United Kingdom; 5 NIHR Health Protection Unit in Gastrointestinal Infections, University of Liverpool, Liverpool, United Kingdom

**Keywords:** Shiga toxin-producing E. coli - STEC, ingredient-based analysis, cohort study, salad leaves

## Abstract

Public Health England was alerted to a national outbreak of Shiga toxin-producing *Escherichia coli* O157 PT34 in July 2016. Early investigations suggested that the likely source was a salad item consumed outside of the home. A number of cases reported consuming meals at a staff canteen (Venue A) and a garden café (Venue B). Both venues shared a common salad supplier. An investigation was undertaken to measure associations between salad items and illness using an 'ingredient-based analysis'. A retrospective case–control study was conducted using an online questionnaire to collect information on menu items consumed at each venue. Chefs at both venues were interviewed to identify ingredients contained within each menu item. Both venues were pooled together for multivariable analysis measuring associations at the ingredient level. Among 203 responses, 24 cases were identified (13 confirmed, two probable and nine possible). Case onsets ranged between 7 and 25 June 2016. Multivariable analysis identified strong evidence that only baby mixed-leaf salad from the common supplier was a vehicle of infection (adjusted odds ratio = 13.1; 95% confidence interval: 1.6–106.5). Identifying the specific salad ingredient associated with illness was made possible by using an ingredient-based analysis. We recommend the increased use of ingredient-based analyses.

## Background

In June 2016, eight cases of Shiga toxin-producing *Escherichia coli* (STEC) serotype O157 phage type (PT) 34 were notified in a 48-hour period in South West England. Nine days after the initial alert, 56 cases of STEC O157 PT34 had been reported across England and Wales, representing a substantial increase compared with expected levels (on average 2.4 cases of STEC O157 PT34 were reported in England and Wales during spring and summer between 1994 and June 2016). Subsequent whole genome sequencing on case isolates revealed that the cases were genetically highly related at the 5-SNP level, confirming that they were outbreak cases.

An outbreak control team was established. Initial investigations included enhanced trawling questionnaires and a case–case study. Results of these investigations suggested that consumption of salad items and eating outside of the home were associated with illness [1].

Two clusters of cases were identified through information in the enhanced questionnaires. A total of eight outbreak cases reported eating either at a staff canteen situated within an office building (Venue A) or at a garden centre café (Venue B). A common wholesale distributor (Distributor B) was found to supply salad ingredients to both venues. Distributor B sourced a proportion of its baby mixed-leaf salad and rocket salad products from a particular supplier (Supplier A). During a parallel trace-back investigation, Supplier A was also identified as a supplier of salad items to 30 venues across the country each associated with at least one case [2].

Food distributors were only able to provide limited information on when specific batches of product were bought and sold. As it was not possible to accurately trace salad products from farm to fork, unconventional epidemiological techniques were required to identify the true source of infection. This study sought to test the hypothesis that eating a salad product that originated from Distributor B was associated with illness.

## Methods

### Study design

A retrospective case–control study was used; people who ate at Venue A or Venue B were pooled together.

### Exposure reference period

The exposure reference period (the period during which contaminated food was served and consumed) differed between the two venues. For venue B, the exposure reference period was taken as the earliest and latest date on which cases reported eating at Venue B: 11 to 19 June 2016. This approach could not be applied to Venue A as this was a staff canteen and therefore multiple visits were likely. The exposure reference period for Venue A was taken as 6 to 17 June 2016; this period was identified by taking 8 days before the earliest case’s symptom onset, known at the time, and the latest case’s symptom onset. The same exposure reference periods were used for cases and controls.

### Study population

The study population consisted of people who ate at Venue A or Venue B during the exposure reference period.

### Recruitment of controls

All employees in the office building who had access to Venue A were identified by building managers and were invited to take part in the study. No such cohort could be identified for Venue B, where the only way to identify controls was to ask cases to identify those they had eaten with. In order to maximise power, no restrictions were placed on the number of controls.

### Case definition

Confirmed cases were defined as those with a reference laboratory-confirmed isolate of *E. coli* O157 PT 34 *eae*+*stx*2+*stx*1− and compatible whole genome sequence (within 5 SNP single linkage cluster with address: 5.156.1329.2502.2965.3081.%), with onset of illness within 8 days of consuming food at Venue A or Venue B during the appropriate exposure reference period.

Probable cases were defined as those with onset of bloody diarrhoea within 8 days of consuming food at Venue A or Venue B during the appropriate exposure reference period.

Possible cases were defined as those with onset of diarrhoea within 8 days of consuming food at the Venue A or Venue B during the appropriate exposure reference period.

Cases were excluded if they reported travelling outside the United Kingdom in the 10 days before symptom onset, or had close contact with other individuals with gastroenteritis in the ten days before symptom onset.

### Data collection

Distributor B provided information on all salad ingredients supplied to Venue A and Venue B. This information was used to produce a list of all salad ingredients commonly supplied to both venues (22 in total). Chefs at Venue A and Venue B were provided with the list of 22 commonly supplied salad ingredients and asked to identify salad ingredients contained in each menu item served during the exposure reference period (76 menu items in total). Menus varied at Venue B but not at Venue A.

An online questionnaire was developed using SelectSurvey.Net (ClassApps, Kansas City, USA); questions included demographic details, details about illness and food items eaten. An email containing a link to the online questionnaire was sent to individuals with available contact details. Phone interviews were conducted to collect information for participants without email addresses.

### Analysis

The numbers of cases and non-cases were reported by venue and described by age, sex and symptoms. The age and sex distribution of cases and non-cases were compared using a Wilcoxon rank-sum test and a chi-squared test, respectively.

To identify ingredient-level exposures, an ingredient-to-menu item lookup table was generated; each row represented an ingredient and each column represented a menu item. The table was populated with 0 (ingredient not included in a menu item) or 1 (ingredient included in a menu item). This lookup table was merged onto the menu item data collected from respondents to give ingredient-level exposure data.

Separate analyses were conducted using a case definition with high specificity (confirmed and probable cases only) and one with high sensitivity (confirmed, probable and possible).

Single variable analysis was undertaken to determine odds ratios (ORs) of the association between exposures (individual ingredients) and outcome. Chi-squared or Fisher’s exact tests were used as appropriate.

Asymptotic logistic regression models were constructed to measure associations between ingredient exposures and being a case; exact models were used where the OR could not be estimated because of a sampling zero. Exposure variables which explained at least 60% of cases and had a crude OR > 2 and a p value < 0.1 during single variable analysis were included in each initial model. Exposures with OR < 1 were removed from the model one at a time starting with those with the largest p value generated from a likelihood ratio test. A variable was added back to the model if removal resulted in a change of more than 20% in the OR for any other variable in the model. We continued this process until we arrived at a final model. The robustness of the final model was verified by adding back variables of interest identified during single variable analysis. The final model was considered robust if no change in interpretation was observed after adding back variables.

## Results

### Questionnaire response

A total of 351 responses were received. We excluded 146 respondents as they reported not eating at either venue during the exposure period of interest. A further two respondents who reported diarrhoea were removed as they failed to provide an onset date and could not be classified as either a case or non-case. There were 203 remaining valid responses.

A total of 186 respondents ate at Venue A and 17 respondents ate at Venue B. Twenty-four respondents were defined as cases: 13 were confirmed cases (five ate at Venue A and eight ate at Venue B), two were probable cases (both ate at Venue A) and nine were possible cases (all ate at Venue A).

### Descriptive epidemiology

Eighteen of 24 cases and 129 of 179 non-cases were female (p = 0.76). The median age of cases and non-cases was 51 years (range: 32–77 years) and 47 years (range: 21–90 years), respectively (p = 0.07) (Table1).

**Table 1:** Demographic data and symptom profile of *Escherichia coli* O157 PT 34 cases and non-cases, South West England, June 2016 (n = 203)

	Cases (n = 24)		Non-cases (n = 179)		Total
Sex					
Female	18	75%	129	72%	147
Male	6	25%	50	28%	56
Total	24	100%	179	100%	203
Age					
Median age (years)	51		47		47
0–19	0	0%	0	0%	0
20–29	0	0%	10	6%	10
30–39	3	13%	37	21%	40
40–49	8	35%	58	33%	66
50–59	7	30%	51	29%	58
60–69	2	9%	14	8%	16
≥ 70	3	13%	4	2%	7
Total	23^a^	100%	174^b^	100%	197
Symptoms					
Diarrhoea	23	96%	10	6%	33
Cramps	21	88%	12	7%	33
Nausea	17	71%	6	3%	23
Bloody stools	13	54%	0	0%	13
Fever	7	29%	6	3%	13
Vomiting	6	25%	4	2%	10

^a^ One case did not report their age.

^b^ Five non-cases did not report their age.

Among those who ate at Venue A, the earliest symptom onset was reported in a possible case on 7 June 2016; with the exception of this case, all possible and probable cases occurred on similar dates to confirmed cases (confirmed cases ranged from 15 and 20 June 2016). Cases peaked between 16 and 18 June 2016. Among those who ate at Venue B, the earliest symptom onset occurred on 15 June 2016; the last symptom onset occurred on 25 June 2016.

## Analytical epidemiology

Single variable analysis using the sensitive case definition found that baby mixed-leaf salad had the largest OR (19.7) and explained 96% of cases (Table 2). Using the specific case definition, the baby mixed-leaf salad and rocket were most strongly associated with being a case, explaining 100% and 80% of cases, respectively.

**Table 2:** Single variable analysis for ingredients consumed at Venue A or Venue B, *Escherichia coli* O157 PT 34 cases and non-cases, South West England, June 2016 (n = 203)

Exposure	Cases (n = 24)			Non-cases (n = 179)			OR	95% CI	p value
	Total ^a^	Exposed	%	Total ^a^	Exposed	%			
Baby mixed-leaf	24	23	96	156	84	54	19.7	3.01–822.99	< 0.01
Red onion	23	15	65	157	48	31	4.3	1.56–12.32	< 0.01
Rocket	23	15	65	145	47	32	3.9	1.43–11.35	< 0.01
Potatoes	24	15	63	157	69	44	2.1	0.81–5.84	0.09
Peppers	24	14	58	157	59	38	2.3	0.89–6.23	0.05
Red cabbage	22	8	36	148	20	14	3.7	1.16–10.73	0.01
Spinach	23	8	35	153	7	5	11.1	2.99–40.80	< 0.01
Bean sprouts	22	5	23	149	15	10	2.6	0.66–8.87	0.09
Mushrooms	24	2	8	157	39	25	0.3	0.03–1.21	0.07

CI: confidence interval; OR: odds ratio.

^a^ Individuals that reported ‘Unsure’ to exposure questions are not reported.

Cases were defined as possible, probable or confirmed cases. Only exposures with p values < 0.1 are presented.

The multivariable analysis found strong evidence of an association between eating baby mixed-leaf salad and being a case using both the sensitive (OR = 13; p < 0.01; 95% CI: 1.62–106.50) and the specific (OR = 7; p = 0.01; 95% CI: 0.87–∞) case definition (Table 3).

**Table 3:** Multivariable analysis for ingredients consumed at the Venue A or Venue B, *Escherichia coli* O157 PT 34 outbreak, South West England, June 2016 (n = 203)

Exposure	Sensitive case definition			Specific case definition		
	aOR	95% CI	p value	aOR	95% CI	p value
Baby mixed leaf	13.15	1.62–106.50	< 0.01	7.23^a^	0.87 – ∞	0.01
Red onion^b^	2.07	0.78–5.50	0.14	NI	NI	NI
Rocket^c^	NI	NI	NI	3.17	0.77 – ∞	0.07

aOR: adjusted odds ratio; CI: confidence interval; NI: not included.

^a^ Median unbiased estimate.

^b^ Not included in the final model built using the specific case definition.

^c ^Not included in the final model built using the sensitive case definition.

Cases were defined using either a sensitive case definition (possible, probable or confirmed case) or a specific case definition (probable or confirmed case, possible cases were removed).

Exposure to a contaminated food item at Venue A most probably occurred between 13 and 15 June 2016 (all confirmed and probable cases and seven of nine possible cases reported eating at Venue A during this time period). Exposure to a contaminated food item at Venue B is likely to have occurred on 11, 14, 18 and 19 June 2016, the dates on which cases reported eating at Venue B.

## Discussion

Our study found that baby mixed-leaf salad supplied by Distributor B was the only ingredient that was independently associated with being a case. Salad leaves are a known vehicle of infection for *E. coli* O157 [3-9], and our findings were consistent with subsequent investigations conducted by the outbreak control team. All other significant exposures identified during single variable analysis were not significant in the multivariable analysis, suggesting confounding.

Use of an ingredient-based analysis allowed us to combine venues into a single study, despite differing menu items. This was possible because of the genetic relatedness of isolates from cases who ate at the two venues and because the venues were supplied by a common distributor, allowing common exposures to be defined between the two venues. Combining venues increased the power of the study, compared with analysing venues separately. Using an ingredient-based analysis and combining venues allowed us to identify, with statistical significance, the specific source of infection. This was essential for the management of this outbreak. The analytical epidemiological association with a specific food item was particularly helpful, in the absence of positive microbiological results from food samples (which were not collected until after the contamination had probably passed). This study helped inform control measures including Supplier A volunteering to suspend the distribution of salad leaves and precautionary media communications advising the public to wash salad leaves before consumption.

Ingredient-based analyses are rarely used in outbreak investigations but, when used, have been successful in identifying the specific source of infection when more conventional methods were unable to identify any source [10-17].

The likelihood of a type I error was reduced in this outbreak thanks to the use of an ingredient-based analysis. There were 76 different menu items served across the two venues containing salad ingredients supplied by Distributor B. By measuring exposures at the ingredient level, the number of exposures to analyse was reduced to 22 salad ingredients.

Use of an ingredient-based analysis may have resulted in more accurate exposure classification compared with a traditional analysis. This is because respondents were more likely to accurately recall which main menu items they had eaten rather than a potentially non-memorable ingredient. Another example of an ingredient-based analysis used to identify a non-memorable ingredient as the source of infection is the outbreak of STEC O104 in Germany in 2011 [11]. In that outbreak, all cases in the ingredient-based study had consumed sprouts but only 25% of cases in the previous case–control study reported eating sprouts. As sprouts were served as garnish or in side salads accompanying main dishes, consumption of this ‘concealed exposure’ was likely to be forgotten. Another example of a concealed exposure identified with an ingredient-based analysis is an outbreak of gastrointestinal illness in California between 1998 and 1999 caused by methomyl-contaminated salt [12].

The source of infection in this outbreak was an ingredient contained within multiple dishes. Use of an ingredient-based analysis resulted in only the specific source of infection, baby-mixed leaf salad, being associated with illness. A traditional analysis may have resulted in multiple menu items being associated with illness, thereby failing to identify the true source of infection.

A potential limitation of the ingredient-based analysis is that ingredients may not be used consistently in dishes, potentially resulting in exposure misclassification. For example, the chef at Venue B could not provide accurate information on which herbs were used in which dishes as this varied from day to day. This limitation did not impact on our study as no herbs were supplied to either venue by the common supplier.

Inaccurate recall was also a potential limitation of this study and of the ingredient-based analysis in general. For respondents, the delay between eating at either venue and completing the study questionnaire ranged from 18 to 38 days (data not shown). For chefs, the delay between preparing food and being interviewed for the study was 32 days for Venue A and 29 days for Venue B (data not shown). Therefore, ingredients may have been used in menu items but not analysed. This inaccurate recall may impact on the strength of associations in either direction.

A general limitation of the ingredient-based analysis is that it is likely to be more resource-intensive than a traditional approach. This is because more information is required from chefs in terms of identifying ingredients included in each menu item. For example, in this study, repeated interviews were conducted with chefs from both venues to ensure accuracy. It is also necessary to match ingredients to each menu item during the analysis.

This outbreak provides further evidence that salad leaves should be considered as a vehicle of infection in STEC and other gastrointestinal infection outbreaks.

Dependent on the context of the outbreak, we recommend the use of ingredient-based analyses. This methodology might be most effective when identifying a specific source of infection and combining multiple sub-clusters of cases into a single study.
